# Transient regulatory T-cell (Treg) depletion with IL-2 diphtheria toxin fusion protein enhances clearance of acute myeloid leukemia by haploidentical natural killer (NK) cells

**DOI:** 10.1186/2051-1426-1-S1-P1

**Published:** 2013-11-07

**Authors:** Veronika Bachanova, Sarah Cooley, Todd Defor, Michael R Verneris, Bin Zhang, David McKenna, Julie Curtsinger, Angela Panoskaltsis-Mortari, Dixie Lewis, Keli Hippen, Philip McGlave, Daniel Weisdorf, Bruce R Blazar, Jeffrey S Miller

**Affiliations:** 1 Blood and Marrow Transplant Program, University of Minnesota Medical School, Minneapolis, MN, USA

## 

Adoptive transfer of haploidentical natural killer (NK) cells can induce remissions in patients with AML. Despite lymphodepleting chemotherapy failure may result from suppression by host regulatory T cells (Treg). We report outcomes of 57 refractory AML patients treated with cyclophosphamide and fludarabine followed by haploidentical NK cells and exogenous IL-2 to facilitate NK proliferation. We augmented Treg lymphodepletion with IL-2-Diphteria toxin fusion protein (IL2DT) in 15 subjects. NK cell-enriched product characteristics are described for three production methods. Day 14 blood donor NK expansion rates were 10% (no IL2DT) versus 30% (with IL2DT) and day 28 leukemia clearance were 25% versus 53% (with IL2DT; p=0.06), suggesting clinical efficacy of IL2DT strategy. Host Treg depletion at day 7 after IL2DT (mean 1% [range 0-4.7%]) correlated with donor NK cell expansion (p=0.01). The only factor correlating with AML clearance was donor chimerism detectable a week after NK cell infusion (mean 49.5%). In vitro, Tregs co-incubated with NK cells exhausted added IL-2 (0.25ng/ml) and suppressed NK cells proliferation. Higher amounts of IL-2 (10ng/ml l) or IL15 (0.5ng/ml ) lead to unaltered NK cell proliferation in vitro even at high NK:Tregs ratios. Future clinical improvements in adoptive cell therapy might include alternate methods of Treg depletion and the use of IL-15 to augment NK cell expansion.

